# Co-Fermented Black Barley and Quinoa Alleviate Hepatic Inflammation via Regulating Metabolic Disorders and Gut Microbiota in Mice Fed with High-Fat Diet

**DOI:** 10.3390/nu17203228

**Published:** 2025-10-15

**Authors:** Fenfen Wei, Huibin Jiang, Chuang Zhu, Lingyue Zhong, Zihan Lin, Yan Wu, Lihua Song

**Affiliations:** Department of Food Science & Technology, School of Agriculture and Biology, Shanghai Jiao Tong University, Shanghai 200240, China; weiff2021@sjtu.edu.cn (F.W.); 122150910120@sjtu.edu.cn (H.J.); zc0324@sjtu.edu.cn (C.Z.); zhonglingyue@sjtu.edu.cn (L.Z.); ceciliah@sjtu.edu.cn (Z.L.); wuy@sjtu.edu.cn (Y.W.)

**Keywords:** co-fermented quinoa and black barley, high-fat diet, hepatic inflammation, hepatic and fecal metabolites, gut microbiota

## Abstract

**Background**: High-fat diet (HFD)-induced hepatic inflammation impairs liver function, promotes fibrosis, and may progress to hepatocellular carcinoma, thereby posing a significant threat to human health. Meanwhile, fermented whole grains have attracted growing attention owing to their diverse beneficial biological properties. **Methods**: In this study, we investigated the effects of co-fermented quinoa and black barley (FG) on HFD-induced chronic hepatic inflammation using male C57BL/6J mice. **Results:** FG intervention significantly attenuated excessive body weight gain and reduced hepatic adipose accumulation in HFD-fed mice. Furthermore, FG alleviated hepatic inflammation by downregulating the transcriptional and protein levels of tumor necrosis factor-α (TNF-α), interleukin-1β (IL-1β), and interleukin-6 (IL-6), as well as the transcriptional levels of toll-like receptor 4 (*Tlr4*), cluster of differentiation 14 (*CD14*), and myeloid differentiation primary response gene 88 (*Myd88*). Metabolomic analysis identified several hepatic and fecal metabolites, such as vitamin A and L-tryptophan, that were upregulated by FG treatment. The strong negative correlation of these metabolites with hepatic inflammatory markers suggests their role as putative mediators of FG’s anti-inflammatory action. Additionally, FG enhanced the relative abundances of probiotic taxa, including *g_Lawsonibacter*, *g_Acetatifactor*, and *s_Bifidobacterium cricetid*, and upregulated the microbial bile acid (BA) biosynthesis pathway. Notably, these enriched probiotics exhibited a positive correlation with the aforementioned fecal metabolites. **Conclusions**: Our findings suggest that FG has the potential to alleviate HFD-induced hepatic inflammation by restoring gut microbiota imbalance and reversing metabolic disorders.

## 1. Introduction

Nowadays, with the advancement of social economy and the improvement of living standards, high-fat diets (HFDs) have become a predominant feature of dietary patterns among a considerable proportion of the population [1]. Prolonged and excessive consumption of HFD induces hepatic lipid accumulation and subsequent low-grade chronic hepatic inflammation [2], which may act as a crucial initiating risk factor for the progression of liver fibrosis, cirrhosis, and hepatocellular carcinoma [3].

Previous studies have demonstrated that excessive fat accumulation exacerbates oxidative stress in the liver, leading to the production of substantial amounts of reactive oxygen species (ROS). These ROS induce damage to hepatocyte membranes and DNA, ultimately promoting cellular apoptosis and necrosis, which in turn triggers inflammatory responses. Under conditions of lipid accumulation and oxidative damage, hepatic stellate cells (HSCs) are activated and secrete a variety of inflammatory cytokines, such as tumor necrosis factor-α (TNF-α) and interleukin-6 (IL-6); this establishes a vicious cycle that further exacerbates hepatocellular injury, perpetuates hepatic dysfunction, and aggravates metabolic imbalance in the liver [4,5,6]. For instance, the liver serves as the primary site for bile acid synthesis. A high-fat diet induces hepatic steatosis and oxidative stress, which in turn causes hepatocyte damage. This damage not only inhibits the transport and secretion of bile acids—resulting in their accumulation in the liver—but also impairs the efficiency of bile acid excretion from the liver, further promoting their retention within the organ. Accumulated bile acid (BA) in the liver subsequently exacerbates hepatic inflammation [7].

Moreover, studies have revealed that gut microbiota dysbiosis induced by HFD may also exert harmful effects on the liver via the gut–liver axis [8]. Specifically, HFD has been shown to alter the gut microbiome and enterohepatic circulation, thereby disrupting bile acid metabolism and impairing liver health; this works in conjunction with the aforementioned factors to drive the onset and progression of chronic low-grade hepatic inflammation [9]. Gut microbiota imbalance—characterized by a high abundance of Firmicutes and a low abundance of Bacteroides—induced by a HFD is significantly positively associated with chronic liver inflammation [10]. Beyond alterations in the microbiota itself, changes in metabolites resulting from microbial dysbiosis are also closely linked to chronic inflammation. For instance, Verena et al. [11] found that the levels of primary bile acids, such as cholic acid (CA) and chenodeoxycholic acid (CDCA), were elevated in the feces of dogs with chronic liver disease, and these levels were associated with the severity and progression of the disease. Notably, chronic liver inflammation is tightly associated with pathological processes such as insulin resistance, which can further exacerbate liver damage and increase the risk of developing various chronic diseases [12]. Therefore, it is imperative to implement strategies for the prevention and mitigation of chronic liver inflammation to ensure effective health management.

In recent years, fermented whole-grain foods have garnered extensive attention, owing to their unique health-promoting properties. The secondary metabolites, produced during the fermentation process, exert a positive effect on ameliorating metabolic disorders [13]. Additionally, probiotics and prebiotics present in fermented whole grains can modulate the composition of the gut microbiota, which is conducive to maintaining gut health [14]. Using multi-omics approaches—including metabolomics, transcriptomics, and microbiomics—our previous studies have demonstrated that fermented grains can ameliorate gut microbiota dysbiosis and metabolic disorders in animal models exposed to a HFD and cigarette smoke [15,16,17]. However, these studies have focused less on their effects on chronic hepatic inflammation caused by a HFD and its association with the gut microbiota.

In our earlier study, a comprehensive chemical analysis demonstrated that co-fermenting quinoa and black barley (FG) yielded a greater enrichment of polyphenols and flavonoids, improved the bioavailability of polyphenols, and strengthened antioxidant activity relative to single-grain fermentation [18]. Therefore, this study was designed to investigate the efficacy of FG in regulating HFD-induced chronic liver inflammation and metabolic disorders in a mouse model. Our research is expected to provide a healthier spectrum of probiotic-fermented whole cereal options.

## 2. Materials and Methods

### 2.1. Materials and Chemical Reagents

Black barley and quinoa were supplied by Feng Zehua Green Ecological Agriculture Cooperatives (Qinghai, China) and Three Rivers Fertile Soil Ecological Agricultural Technology Co., Ltd. (Qinghai, China), respectively. *Lactobacillus kisonensis* (strain JCM15041) was acquired from the State Key Laboratory of Bioreactor Engineering, East China University of Science and Technology. Enzyme-linked immunosorbent assay (ELISA) kits targeting tumor necrosis factor-alpha (TNF-α, catalog no. CSB-E04744m), interleukin-1β (IL-1β, catalog no. CSB-E08054m), and interleukin-6 (IL-6, catalog no. CSB-E04639m) were procured from Cusabio^®^ (Wuhan, China). All other chemical reagents utilized in this study were sourced from Sinopharm Chemical Reagent Co., Ltd. (Shanghai, China).

### 2.2. Preparation of FG with Lactobacillus

The co-fermentation of whole black barley and quinoa was performed following a previously established protocol [18]. In detail, whole black barley and quinoa were first crushed, and the resulting grain powder was sieved through an 80-mesh sieve. Subsequently, black barley powder and quinoa powder were blended at a weight ratio (*w*/*w*) of 2.4:1. Purified water was added to this mixed grain powder, following a solid-to-liquid mass ratio (*m*/*m*) of 1:8.9. To saccharify the mixture, thermostable α-amylase was introduced at a dosage of 10 U/g of the mixture, and the system was maintained at a constant temperature of 70 °C. This saccharification step was continued until the soluble sugar content in the mixture reached 12%. Afterward, the enzymatic hydrolysate of the mixed grains was subjected to sterilization and cooling. 2% (*v*/*v*) of *Lactobacillus* culture (containing 10^8^ live bacteria) was inoculated into the hydrolysate and the inoculated mixture was then incubated at 30 °C for a fermentation period of 36 h. At the end of the co-fermentation process, the number of live bacteria in the fermented grain product was approximately 5 × 10^8^ CFU/mL. The nutritional composition, including protein, dietary fiber, and polyphenols, was profiled in our previous study [18].

### 2.3. Animals and Treatments

To ensure the repeatability and stability of experimental data, a total of 40 six-week-old male C57BL/6J mice (purchased from SLAC Laboratory Animal Co., Ltd., Shanghai, China) were first allowed to adapt to the housing environment for one week. The mice were housed under controlled conditions: a constant temperature of 25 °C and a 12 h light/12 h dark cycle. After the adaptation period, the mice were randomly assigned to four groups according to body weight, with 10 mice in each group, and 5 mice in each cage. All the mice had free access to water and food, and the feeds were granulated. The grouping and intervention schemes were as follows: normal control group (NC): fed a standard control diet and administered sterile water; high-fat diet group (HFD): fed a high-fat diet (FB-C45HFDC1, Wuxi Fanbo Biotechnology Co., Ltd., Wuxi City, Jiangsu Province, China) and administered sterile water; high-fat diet + FG treatment group (HFG): fed the high-fat diet and administered FG at a dose of 10 mL/kg. The components of FG were characterized as reported in our previous study [18]; high-fat diet + *Lactobacillus* treatment group (HFL): fed the high-fat diet and administered Lactobacillus at a dose of 10 mL/kg. The composition of the standard control diet was as follows: 58.75% carbohydrates, 18% crude protein, 4% crude fat, 5% crude fiber, 10% water, 1.5% calcium, 1% total phosphorus, 0.82% lysine, 0.53% methionine + cysteine, and 0.4% sodium chloride. The high-fat diet was prepared by mixing the standard control diet (46.25%, *wt*/*wt*) with additional components: 18% lard (*wt*/*wt*), 20% sucrose (*wt*/*wt*), 10% casein, 1.25% cholesterol, 0.5% cholate, 0.5% premixed multivitamins, 1.5% multimineral, and 2% maltodextrin. All groups received their respective interventions for a continuous period of 10 weeks. During the intervention, the body weight and food intake of each mouse were monitored twice weekly. At the end of the intervention, the mice were deeply anesthetized with 2% isoflurane until unresponsive to toe pinch stimulation and then sacrificed by cervical dislocation. All animal experimental procedures were reviewed and approved by the Animal Ethics Committee of Shanghai Jiao Tong University (A2023020). Technicians involved in all tests were blinded to the group allocations of all samples.

#### 2.3.1. Analysis of Organs Index and Colon Length

The indexes of the liver, gonadal adipose tissue, and perirenal adipose tissue were determined by the following formula:

organ index (%) = (mass of the organ/body mass of the mouse) × 100%.

#### 2.3.2. Hepatic Hematoxylin–Eosin (HE) Staining Assay

First, liver tissue samples were fixed in 4% paraformaldehyde. After fixation, the tissues were dehydrated using gradient concentrations of ethanol. Subsequently, the dehydrated liver tissues were subjected to HE staining following standard protocols. For pathological analysis, the prepared HE-stained tissue sections were observed and analyzed using an optical microscope equipped with an imaging system (LEICA TCS SP8, Wetzlar, Germany) to acquire images of histological features.

#### 2.3.3. Quantification of Inflammatory Cytokines Using Enzyme-Linked Immunosorbent Assay (ELISA)

The concentrations of inflammatory cytokines, encompassing tumor necrosis factor-α (TNF-α), interleukin-1β (IL-1β), and interleukin-6 (IL-6), in liver tissue were quantified utilizing commercially ELISA kits according to the manufacturer’s protocols. Briefly, 70 mg of mouse liver tissue samples were each homogenized in 700 μL of 1 × PBS solution, and the homogenates were then placed at −20 °C overnight. After undergoing two freeze–thaw cycles, the tissue homogenates were centrifuged at 4 °C for 5 min at a speed of 7300 rpm. Subsequently, the absorbance of the resulting supernatants was measured at a wavelength of 450 nm, and the concentrations of the target inflammatory cytokines were calculated in accordance with the manufacturers’ protocols.

#### 2.3.4. RNA Extraction and Quantitative (q) Real-Time Polymerase Chain Reaction (qPCR)

Total RNA was isolated from liver tissue using the MiniBEST Universal RNA Extraction Kit (catalog number: 9767, Takara, Kusatsu, Japan). The concentration of the extracted RNA was measured via the Nanodrop 2000c spectrophotometer (Thermo Scientific, Waltham, MA, USA), while the quality and purity of RNA were assessed through agarose gel electrophoresis. For cDNA synthesis, 1 μg of total RNA from each sample was reverse-transcribed using the PrimeScript™ RT Reagent Kit (catalog number: RR037A, Takara, Japan). The qPCR reaction mixture was prepared with the TB Green^®^ Premix Ex Taq™ kit (catalog number: RR420A, Takara), and the qPCR reaction was conducted on the SLAN-965 Real-Time PCR system (Hongshi Medical Technology Co., Ltd., Shanghai, China). All experimental procedures were carried out in strict compliance with the instructions provided by the respective kit and instrument manufacturers. The relative mRNA expression levels were normalized to the housekeeping gene β-actin, and the final relative expression values were calculated using the 2^−ΔΔCT^ method. The primers required for qPCR were synthesized by Sangon Biotech Co., Ltd. (Shanghai, China), and their specific sequences are presented in Table 1.

#### 2.3.5. Untargeted Metabolomic Analysis of Fecal and Hepatic Metabolites Using Ultra-Performance Liquid Chromatography Quadrupole Time-of-Flight Mass Spectrometry (UPLC-QTOF-MS^E^)

The metabolomics analysis was performed in line with our previously established method [17]. Briefly, 50 mg of mouse feces was mixed with 200 μL of methanol–acetonitrile (1:1, *v*/*v*), while 50 mg of mouse liver samples was mixed with 500 μL of methanol–acetonitrile–water (2:2:1, *v*/*v*/*v*). After homogenization, the mixtures were incubated at −20 °C overnight, with vertexing every 30 min within the first 2 h. Subsequently, the fecal and liver homogenates were centrifuged at 4 °C (12,000 rpm/min for 20 min).

Metabolites were analyzed via UPLC-QTOF-MS^E^ (Acquity UPLC I-class & Vion IMS QTOF MS, Waters Co., Milford, MA, USA) equipped with an ACQUITY UPLC BEH C18 column (100 mm × 2.1 mm, 1.7 μm, Waters Co., USA). The column temperature was set at 45 °C, and the injection volume was 1 μL. The mobile phase consisted of phase A (0.1% formic acid in water) and phase B (0.1% formic acid in acetonitrile), with a flow rate of 0.4 mL/min. The gradient elution program was as follows: 0–1 min, 5–20% B; 1–2.5 min, 20–40% B; 2.5–9 min, 40–100% B; 9–12 min, 100% B; 12–12.5 min, 100–5% B; 12.5–14.5 min, 5% B.

Mass spectrometry parameters were set as follows: electrospray ionization (ESI) was used in both positive (ESI^+^) and negative (ESI^−^) modes, with capillary voltages of 1.5 kV (ESI^+^) and 1 kV (ESI^−^). For MS^E^ experiments, collision energy was set at 6 eV (low energy) and 20–45 eV (high energy). Argon (99.999%) served as the induced dissociation gas, and nitrogen (>99.5%) as the cone and desolation gas. The flow rates of cone gas and desolation gas were 50 L/h and 900 L/h, respectively; the source temperature was 115 °C, and the cone voltage was 40 V.

Raw data were imported into Progenesis QI v2.3 software (Waters Co., USA) for peak alignment, peak picking, and deconvolution. Metabolites were identified using online databases (Human Metabolome Database, LIPID MAPS, Metlin). Ion intensity normalization was performed via MetaboAnalyst; variable importance in projection (VIP) values were calculated using the partial least squares discrimination analysis (PLS-DA) model, and *p* values via one-way analysis of variance (ANOVA). Differential metabolites were selected with VIP > 1.00 and *p* < 0.05, and all were subjected to pathway analysis in MetaboAnalyst based on the Kyoto Encyclopedia of Genes and Genomes (KEGG) database.

#### 2.3.6. Metagenomic Analysis

Total microbial genomic DNA was isolated from samples using the OMEGA Mag-Bind Soil DNA Kit (catalog no. M5635-02, Omega Bio-Tek, Norcross, GA, USA), following the manufacturer’s instructions. DNA quantity was assessed via a Qubit™ 4 Fluorometer (catalog no. Q32856, Invitrogen, Carlsbad, CA, USA), while DNA quality was determined by agarose gel electrophoresis.

Microbial DNA was processed to construct metagenome shotgun sequencing libraries (insert size: 400 bp) using the Illumina TruSeq Nano DNA LT Library Preparation Kit. Each library was sequenced on the Illumina NovaSeq platform (Illumina, San Diego, CA, USA) with the PE150 strategy, conducted at Personal Biotechnology Co., Ltd. (Shanghai, China). Raw sequencing reads were processed to obtain quality-filtered reads for subsequent analysis. Sequencing adapters were removed using Cutadapt (v1.2.1), and low-quality reads were trimmed via a sliding-window algorithm in fastp. Reads were aligned to the Mus musculus host genome using BMTagger to eliminate host contamination.

After obtaining quality-filtered reads, taxonomic classification of metagenomic sequencing reads from each sample was performed using Kraken2. Generated contigs (length > 300 bp) were pooled and clustered with mmseqs2; the lowest common ancestor taxonomy of non-redundant contigs was obtained by aligning them to the NCBI-nt database via mmseqs2. To evaluate gene abundances, high-quality reads from each sample were mapped to predicted gene sequences using salmon, and abundance values in metagenomes were normalized using copy per kilobase per million mapped reads (CPM). The function of non-redundant genes was annotated via mmseqs2, based on the Kyoto Encyclopedia of Genes and Genomes (KEGG) protein databases.

### 2.4. Statistical Analysis

Statistical analysis was carried out using GraphPad Prism 8.0 software (GraphPad Software, Boston, MA, USA). The data were evaluated with analysis of variance (ANOVA) for multiple comparisons, followed by Tukey’s Honestly Significant Difference (HSD) multiple comparison test. *p* value < 0.05 was regarded as indicating a significant difference. All data were presented in the form of mean ± standard deviation (SD).

## 3. Results

### 3.1. Co-Fermented Quinoa and Black Barley (FG) Prevented High-Fat Diet (HFD)-Induced Obesity in Mice

**Figure 1:**
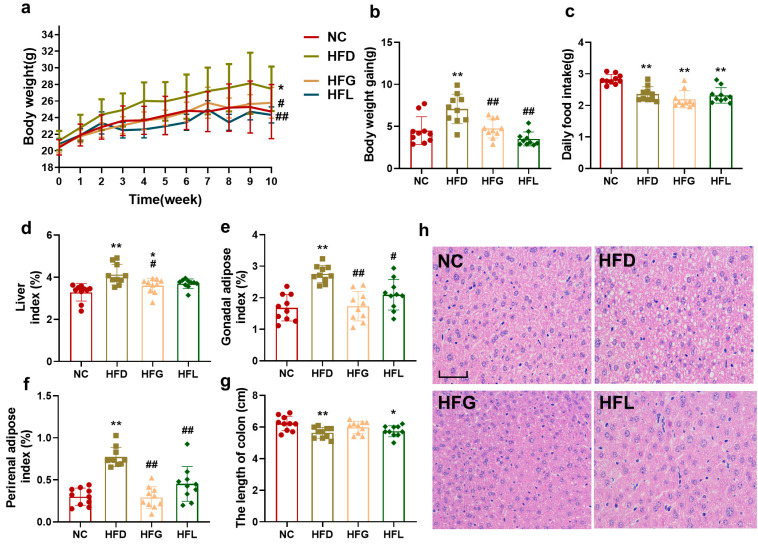
FG reduced body weight as well as liver and adipose tissue index in HFD-fed mice. (**a**) Body weight, (**b**) body weight gain, (**c**) daily food intake, (**d**) liver index, (**e**) gonadal adipose index, (**f**) perirenal adipose index, (**g**) the length of the colon, (**h**) HE staining of hepatic tissue (200×; *n* = 5, scale bar = 50 µm. All data are shown as the mean ± SD (*n* = 10). * p < 0.05 and ** p < 0.01 vs. the NC group, and *^#^ p* < 0.05 and *^##^ p* < 0.01 vs. the HFD group.

As illustrated in Figure 1a, b, following a 10-week feeding period, the HFD group exhibited a significant increase in body weight (27.48 g), which was significantly higher than that of the NC group (24.73 g) (*p* < 0.05). The average body weight of the HFG group was 25.78 g, representing a significant reduction compared with the HFD group (*p* < 0.05); the HFL group showed the same trend as the HFG group. Additionally, the weight gain values of the NC, HFD, HFG, and HFL groups were 4.83 g, 6.77 g, 4.76 g, and 3.52 g, respectively. Notably, the HFG and HFL groups had significantly lower weight gain than the HFD group (*p* < 0.01). Regarding daily food intake, mice in the three high-fat diet-fed groups (HFD, HFG, HFL) had a significantly lower daily food intake compared with those in the NC group (*p* < 0.01, Figure 1c).

Long-term consumption of high-fat diets typically leads to excessive accumulation of adipose tissue. The organ indexes of the liver, gonadal adipose tissue, and perirenal adipose tissue in the HFD group were 4.12, 2.76, and 0.77, respectively—all of which were significantly higher than those in the NC group (liver: 3.28; gonadal adipose tissue: 1.68; perirenal adipose tissue: 0.29) (*p* < 0.01, Figure 1d–f). After intervention with FG, these organ indexes were significantly lower than those in the HFD group (*p* < 0.01 or *p* < 0.05); in contrast, intervention with *Lactobacillus* alone failed to reduce the liver index. Furthermore, the colon length in the HFD group (5.64 cm) was significantly shorter than that in the NC group (6.22 cm). FG intervention showed a modest preservation of colon length (5.98 cm), which, while not statistically significant (*p* > 0.05) compared to the HFD and NC groups, nevertheless demonstrated a more favorable effect than Lactobacillus intervention alone (Figure 1g).

Hepatic HE-stained pathological sections are presented in Figure 1h. In the NC group, hepatocytes and their nuclei were of normal size, with distinct intercellular boundaries and no obvious lipid vacuoles were observed. In contrast, the HFD group showed a prominent presence of large lipid vacuoles in the liver, accompanied by hepatocyte enlargement, abnormal cellular morphology, and indistinct boundaries—features consistent with hepatic steatosis. In the HFG group and HFL groups, a number of clear small lipid droplets were observed both inside hepatocytes and in the spaces between hepatocytes; however, compared with the HFD group, the number of lipid droplets in these two groups was reduced, and their volume was also notably smaller.

### 3.2. FG Reduced Hepatic Inflammation in HFD-Fed Mice

**Figure 2:**
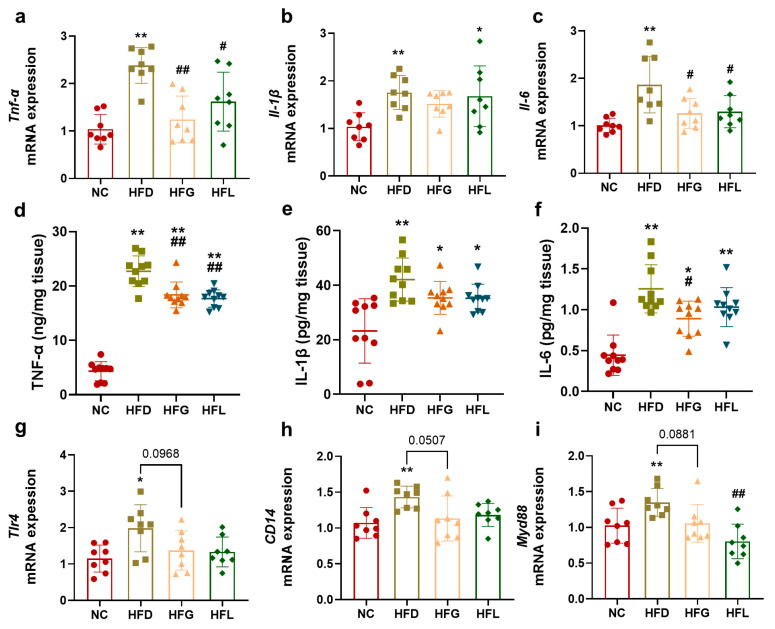
FG alleviated hepatic inflammation in HFD-fed mice. (**a**–**c**) The hepatic mRNA expression of *Tnf-α*, *Il-1β*, and *Il-6* (*n* = 8). (**d**–**f**) The hepatic concentration of TNF-α, IL-1β, and IL-6 (**g**–**i**) (*n* = 10). The hepatic mRNA expression of *Tlr4*, *CD14*, and *Myd88* (*n* = 8), (**g**–**i**) The hepatic mRNA expression of *Tlr4*, *CD14*, and *Myd88* (*n* = 8). All data are shown as the mean ± SD. * p < 0.05 and ** p < 0.01 vs. the NC group, and *^#^ p* < 0.05 and *^##^ p* < 0.01 vs. the HFD group.

It is well established that excessive consumption of a high-fat diet elevates the levels of pro-inflammatory cytokines (including TNF-α, IL-1β, and IL-6) in multiple organs, with the liver being particularly affected. In the present study, the mRNA expression levels of *Tnf-α*, *Il-1β*, and *Il-6* were significantly higher in the HFD group than in the NC group (*p* < 0.01). However, administration of FG significantly downregulated the expression of *Tnf-α* (*p* < 0.01) and *Il-6* (*p* < 0.05); although *Lactobacillus* intervention alone exerted a similar inhibitory effect, its efficacy was lower than that of FG intervention (Figure 2a–c).

Furthermore, ELISA was used to determine the concentrations of these pro-inflammatory cytokines in liver tissue. The concentrations of TNF-α, IL-1β, and IL-6 in the HFD group were 22.71 ng/mg tissue, 42.09 pg/mg tissue, and 1.26 pg/mg tissue, respectively—all significantly higher than those in the NC group (TNF-α: 4.32 ng/mg tissue; IL-1β: 23.21 pg/mg tissue; IL-6: 0.44 pg/mg tissue; *p* < 0.01). In contrast to the HFD group, FG intervention led to a significant reduction in TNF-α (*p* < 0.01) and IL-6 concentrations (*p* < 0.05). *Lactobacillus* intervention alone significantly decreased TNF-α concentration (*p* < 0.01) but had a limited effect on the concentrations of IL-1β and IL-6 (*p* > 0.05, Figure 2d–f). Notably, the TLR4/CD14/MyD88 pathway regulates the production of pro-inflammatory cytokines and drives chronic inflammation. We also analyzed the hepatic mRNA expression levels of *Tlr4*, *CD14*, and *Myd88*. As illustrated in Figure 2g–i, compared to the NC group, the HFD group exhibited significantly higher mRNA expression levels of *Tlr4* (*p* < 0.05), *CD14*, and *Myd88* (*p* < 0.01); in contrast, the HFG group showed numerically lower mRNA expression of these genes compared with the HFD group, though no statistical significance was observed (*p* > 0.05). *Lactobacillus* intervention alone significantly inhibited the mRNA expression of Myd88 (*p* < 0.01).

### 3.3. FG Reversed HFD-Induced Hepatic Metabolites Dysbiosis

**Figure 3:**
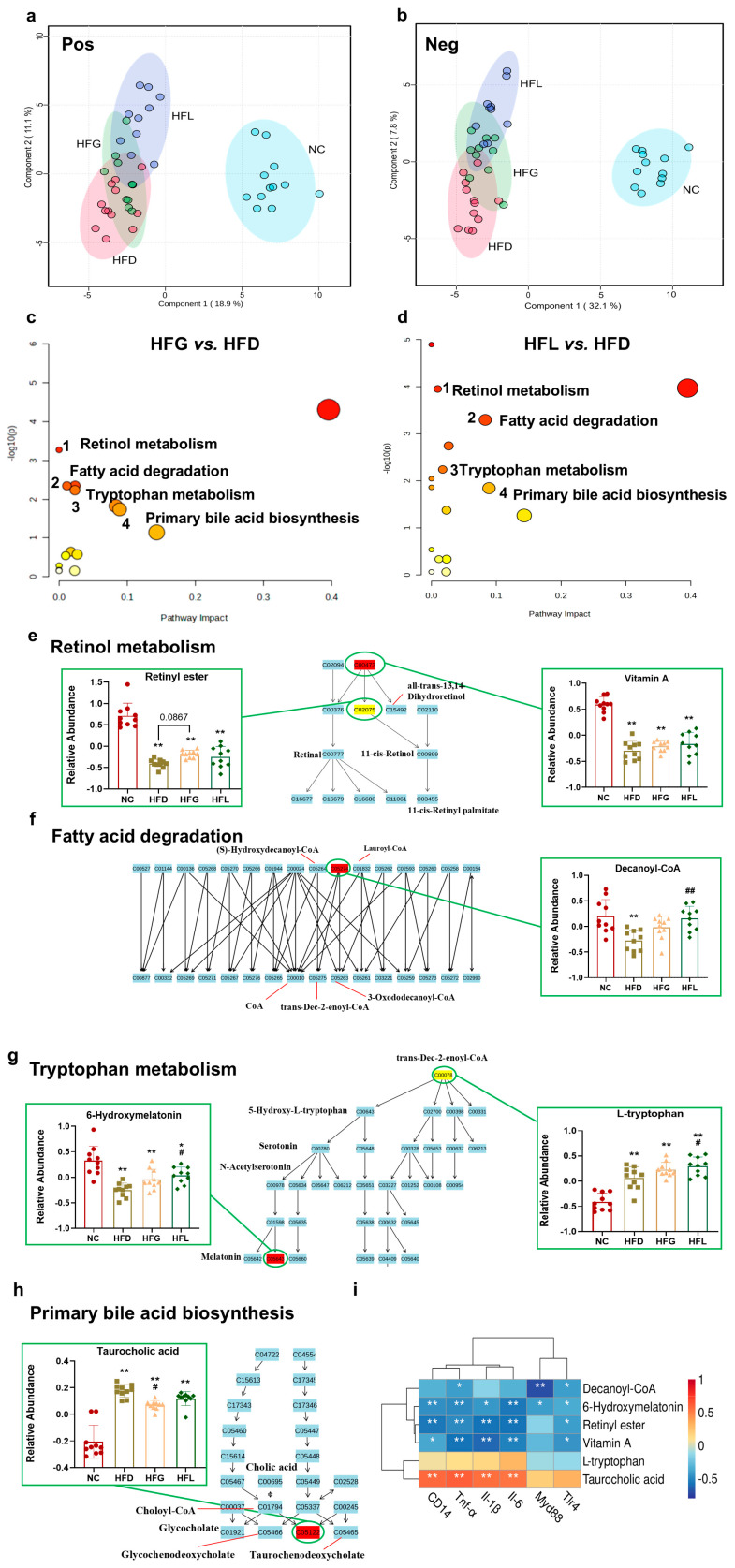
FG partially reversed the HFD-induced alterations in the hepatic metabolic profile. (**a**) Partial least squares discriminant analysis (PLS-DA) plot of liver metabolomic profiles of mice in ESI^+^. (**b**) PLSDA plot of liver metabolomic profiles of mice in ESI^−^. (**c**) Integrative plot of liver metabolites and the relevant pathways (HFG vs. HFD). (**d**) Integrative plot of liver metabolites and the relevant pathways (HFL vs. HFD). (**e**–**h**) Liver differential metabolites within related metabolic pathways. (**i**) Correlation analysis between relative abundances of differential metabolites and inflammatory cytokines in mice liver. All data are shown as the mean ± SD (*n* = 10). * p < 0.05 and ** p < 0.01 vs. the NC group, and *^#^ p* < 0.05 and *^##^ p* < 0.01 vs. the HFD group.

Given the protective effects of FG on the liver—including reduced hepatic lipid accumulation and inflammatory levels—and the critical role of the liver in metabolism, an untargeted metabolomics analysis of liver tissue was performed by using UPLC-Q-TOF-MS^E^. Partial least squares discrimination analysis (PLS-DA) score plots for each group are presented in Figure 3a,b. These score plots revealed distinct clustering between the NC group and the three HFD-fed groups; notably, the HFG and HFL groups showed a tendency to diverge from the HFD group.

A total of 881 metabolites were detected, with 484 identified in the positive ion mode (ESI^+^) and 397 in the negative ion mode (ESI^−^). Overall, 77 differential hepatic metabolites were identified. As summarized in Table 2, FG intervention normalized 17 of these differential hepatic metabolites compared with the HFD group. These results suggested that interventions with FG and *Lactobacillus* may alleviate HFD-induced hepatic metabolic disturbances.

Furthermore, KEGGpathway enrichment analysis was performed on the differential metabolites listed in Table 2, leading to the identification of 4 significantly altered metabolic pathways. These pathways included retinol metabolism, fatty acid degradation, tryptophan metabolism, and primary bile acid biosynthesis (Figure 3c,d). FG intervention up-modulated the following pathway-specific metabolites: retinyl ester and vitamin A in retinol metabolism, decanoyl-CoA in fatty acid degradation, 6-hydroxymelatonin and L-tryptophan in tryptophan metabolism pathway; and down-regulated taurocholic acid (*p <* 0.05) in bile acid biosynthesis (Figure 3e–h). Similarly, intervention with *Lactobacillus* alone exhibited the same corrective trend toward these key metabolites. These observations clarify the potential roles of specific metabolites in distinct biochemical pathways and highlight FG’s regulation on hepatic metabolism.

To elucidate the mechanistic link between hepatic metabolites and inflammation in HFD-induced injury, we examined the correlations between metabolite levels and the expression of inflammatory cytokines (Figure 3i). The relative abundances of retinyl ester, vitamin A, decanoyl-CoA, and 6-hydroxymelatonin were significantly negatively correlated with the expression of *Tlr4*, *CD14*, *Tnf-α*, *Il-1β*, and *Il-6*. Conversely, the relative abundance of taurocholic acid showed a significant positive correlation with the expression of *Tnf-α*, *Il-1β*, *CD14*, and *Il-6*.

### 3.4. FG Rescued the Gut Microbiome Dysbiosis Caused by HFD

**Figure 4:**
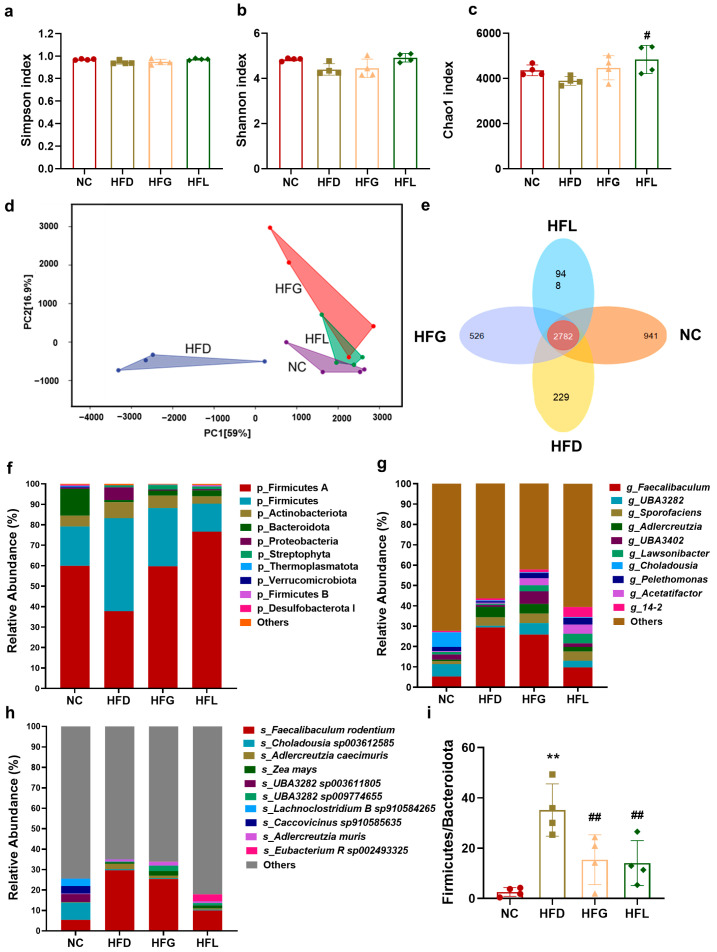

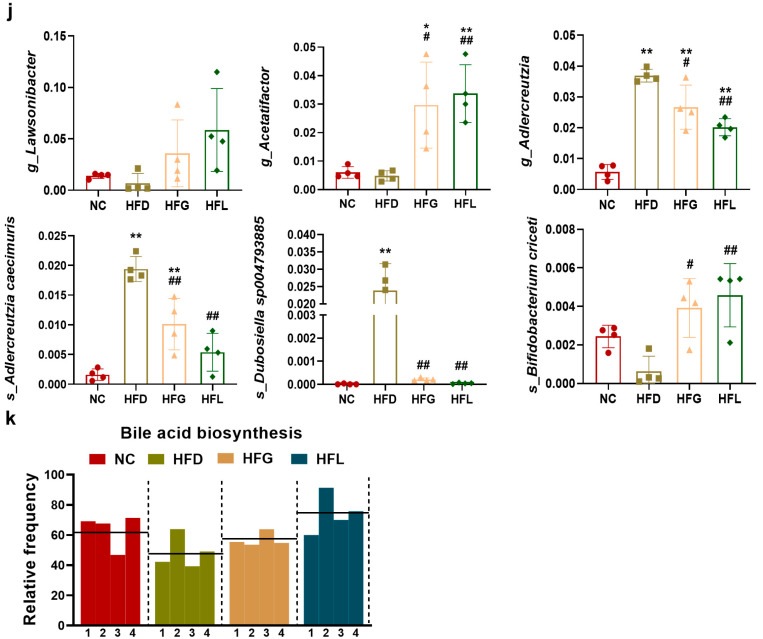
FG reversed HFD-induced gut dysbiosis. (**a**–**c**) α-diversity of Simpson, Shannon and Chao1 indexes. (**d**) Principal component analysis (PCA) plots of beta-diversity. (**e**) Venn diagram based on the species levels of microbiomes. (**f**) Relative abundance of major microbial phyla in the different groups of mice. (**g**) Relative abundance of major microbial genus in the different groups of mice. (**h**) Relative abundance of major microbial species in the different groups of mice. (**i**) Relative abundance of Firmicutes/Bacteroidetes (F/B). (**j**) Comparative analysis of the relative abundance of *g_Lawsonibacter*, *g_Acetatifactor*, *g_Adlercreutzia*, *s_Adlercreutzia caecimuris*, *s_Dubosiella sp004793885*, and *s_Bifidobacterium criceti*. (**k**) The microbial relative frequency of bile acid (BA) biosynthesis. All data are shown as the mean ± SD (*n* = 4). * p < 0.05 and ** p < 0.01 vs. the NC group, and *^#^ p* < 0.05 and *^##^ p* < 0.01 vs. the HFD group.

Diet is a major environmental factor influencing the composition of the gut microbiome. High-fat diets often induce gut microbiome dysbiosis and are associated with various chronic diseases. Therefore, metagenomic sequencing was performed on fecal samples from the mice of each group in this study. Mice in the HFD group exhibited a decreasing trend in alpha diversity, as evidenced by lower Simpson, Shannon and Chao1 indexes (Figure 4a–c). Compared to the HFD group, FG and *Lactobacillus* (*p* < 0.05) intervention increased the Chao1 index—a finding likely attributed to the more pronounced effect of direct probiotic administration on gut microbes.

Principal component analysis (PCA) plots for beta diversity were generated using the Bray–Curtis distance to assess intergroup differences. These plots effectively illustrated variations in the community composition of samples and highlighted distinctions among the four groups (Figure 4d). A Venn diagram revealed that the NC, HFG, and HFL groups harbored 941, 526, and 948 unique species, respectively, whereas the HFD group contained only 229 unique species (Figure 4e). This observation suggests that species diversity was reduced in the HFD group. However, following intervention with FG or *Lactobacillus*, the number of specific species was increased—indicating that these interventions reversed HFD-induced reductions in microbial diversity.

To further explore the effects of FG treatment on the gut microbiota, we analyzed the distribution of gut microbes at the phylum, genus, and species levels. At the phylum level, HFD-fed mice showed increased relative abundance of Firmicutes and decreased relative abundance of Bacteroidetes (Figure 4f). The HFG and *Lactobacillus* interventions both reversed this shift by significantly lowering the Firmicutes-to-Bacteroidetes (F/B) ratio. Specifically, the F/B ratio was significantly lower in the HFG group (14.30 ± 8.92) than in the HFD group (35.91 ± 9.13; *p* < 0.01). Treatment with *Lactobacillus* alone also resulted in a significantly reduced F/B ratio (11.38 ± 9.82; *p* < 0.01). (Figure 4i).

At the genus level (Figure 4g, j), the HFG group exhibited a significantly enriched relative abundance of *g_Acetatifactor* (*p* < 0.05); even more notably, the HFL group showed an extremely significantly increased relative abundance of *g_Acetatifactor* (*p* < 0.01). Additionally, the relative abundance of *g_Adlercreutzia* was significantly increased in the HFD group, whereas FG interventions significantly inhibited the proliferation of this genus (*p* < 0.05) and *Lactobacillus* intervention also showed an extremely significantly inhibitory effect (*p* < 0.01). At the species level (Figure 4h,j), the HFD group exhibited significantly increased relative abundances of *s_Adlercreutzia caecimuris* (*p* < 0.01) and *s_Dubosiella sp004793885* (*p* < 0.01). In contrast, the abundances of these two species were significantly reduced in the HFG and HFL groups (*p* < 0.01), with their values being more similar to those in the NC group. Moreover, FG interventions also significantly increased the abundance of *s_Bifidobacterium criceti* (*p* < 0.05)*,* and *Lactobacillus* intervention alone showed an extremely significantly increased relative abundance of *s_Bifidobacterium criceti* (*p* < 0.01).

Furthermore, we analyzed microbial functions based on the KEGG database. The bile acid biosynthesis pathway was significantly downregulated in the HFD group compared with the NC group (Figure 4k); conversely, this pathway was significantly upregulated in the HFG and HFL groups compared with the HFD group.

### 3.5. FG Partially Reversed HFD-Induced Fecal Metabolites Disorder

**Figure 5:**
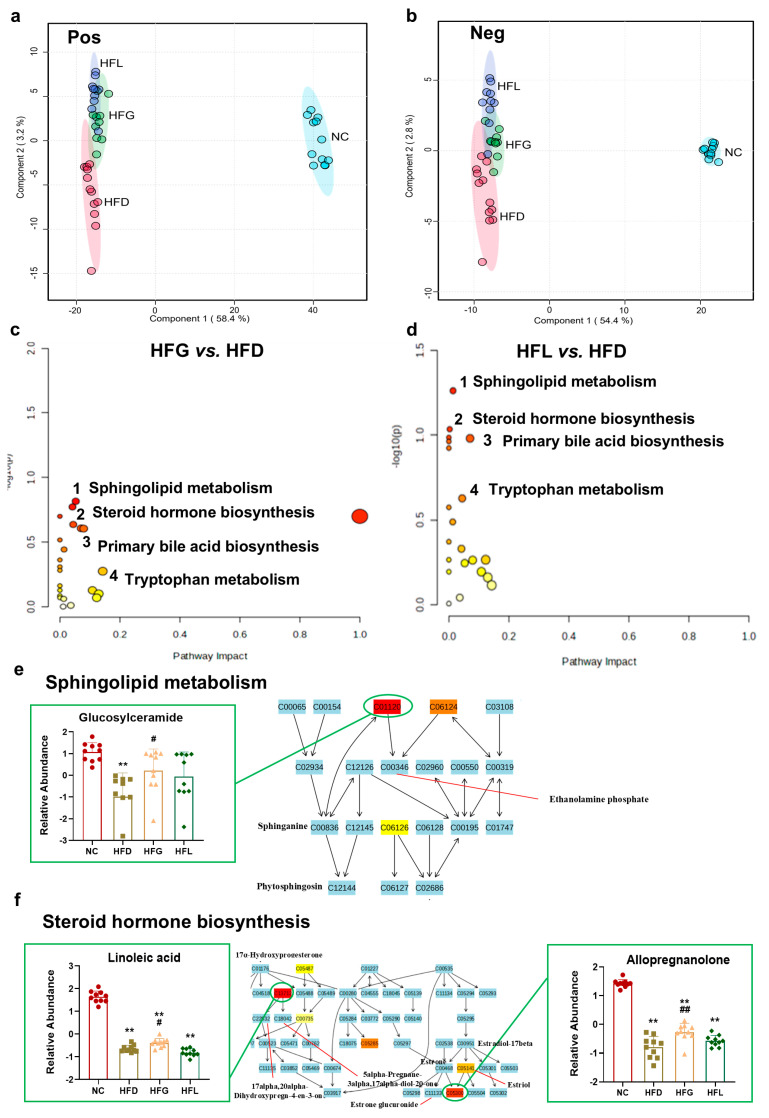

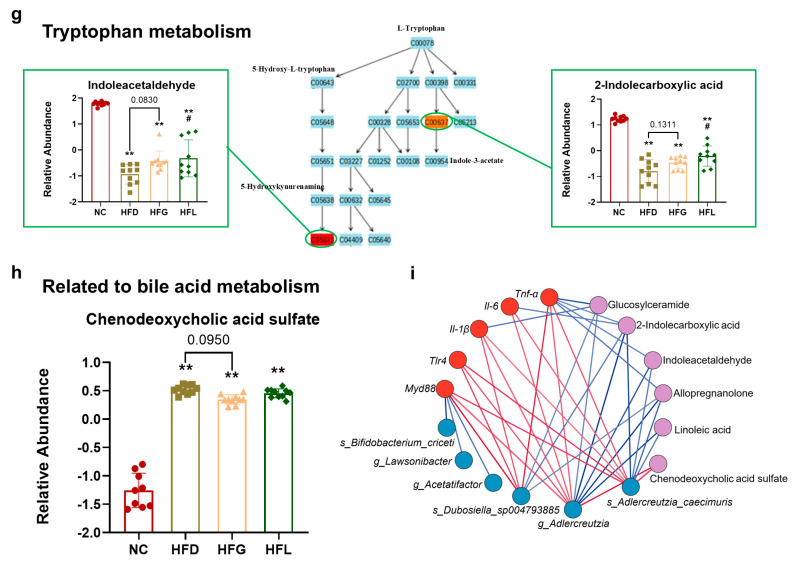
FG partially reversed the HFD-induced alterations in the fecal metabolic profile. (**a**) Partial least squares discriminant analysis (PLSDA) plot of fecal metabolomic profiles of mice in ESI^+^. (**b**) PLSDA plot of fecal metabolomic profiles of mice in ESI^−^. (**c**) Integrative plot of fecal metabolites and the relevant pathways (HFG vs. HFD). (**d**) Integrative plot of liver metabolites and the relevant pathways (HFL vs. HFD). (**e**–**h**) Fecal differential metabolites within related metabolic pathways. (**i**) Network analysis among gut microbiota, fecal metabolites, and hepatic inflammatory cytokines; the red line represents a positive correlation and the blue line represents a negative correlation. All data are shown as the mean ± SD (*n* = 10). ** p < 0.01 vs. the NC group, and *^#^ p* < 0.05 and *^##^ p* < 0.01 vs. the HFD group.

Given the beneficial effects of FG on HFD-induced gut microbiome dysbiosis, we further analyzed fecal metabolites using UPLC-Q-TOF-MS^E^. PLS-DA score plots for each group are presented in Figure 5a, b. These score plots revealed distinct clustering between the NC group and the three HFD-fed groups; notably, the HFG and HFL groups showed a tendency to diverge from the HFD group.

A total of 3665 fecal metabolites were detected, with 2807 identified in the positive ion mode (ESI^+^) and 858 in the negative ion mode (ESI^−^). Overall, 221 differential fecal metabolites were identified. As detailed in Table 3, FG intervention normalized 20 of these differential fecal metabolites compared with the HFD group. These results suggested that interventions with FG and *Lactobacillus* may alleviate HFD-induced fecal metabolic disturbances.

Furthermore, KEGG pathway enrichment analysis was performed on the differential metabolites listed in Table 3, leading to the identification of 4 significantly altered metabolic pathways. These pathways included sphingolipid metabolism, steroid hormone biosynthesis, primary bile acid biosynthesis, and tryptophan metabolism (Figure 5c,d). FG intervention up-modulated the following pathway-specific metabolites: glucosylceramide in sphingolipid metabolism (*p* < 0.05); linoleic acid (*p* < 0.05) and allopregnanolone (*p* < 0.01) in steroid hormone biosynthesis; indoleacetaldehyde and 2-indolecarboxylic acid in tryptophan metabolism; and down-regulated chenodeoxycholic acid sulfate in bile acid biosynthesis (Figure 5e–h). Similarly, intervention with *Lactobacillus* alone exhibited the corrective trend toward these key metabolites. These observations clarify the potential roles of specific metabolites in distinct biochemical pathways and highlight FG’s regulation on fecal metabolism.

To further clarify how gut bacteria and metabolites may influence HFD-induced hepatic inflammation, we analyzed the statistical correlations between gut microbiota, fecal metabolites, and hepatic inflammatory markers (Figure 5i). Notably, the abundance of *g_Adlercreutzia* and *s_Adlercreutzia caecimuris* was positively correlated with chenodeoxycholic acid sulfate; this metabolite, in turn, showed a significant positive correlation with the expression of *Tlr4* and *Il-6.* In contrast, *s_Dubosiella sp004793885*, *g_Adlercreutzia*, and *s_Adlercreutzia caecimuris* showed negative correlations with the following metabolites: indoleacetaldehyde, 2-indolecarboxylic acid, and allopregnanolone. These three metabolites (indoleacetaldehyde, 2-indolecarboxylic acid, and allopregnanolone) exhibited significant negative correlations with the expression of *Tlr4*, *Il-6*, *Tnf-α*, and *Il-1β*.

## 4. Discussion

### 4.1. FG Ameliorated Hepatic Chronic Inflammation Induced by Long-Term HFD

Prolonged excessive consumption of a high-fat diet can induce metabolic disorders and gut microbiota dysbiosis, which in turn lead to elevated levels of chronic hepatic inflammation and a series of health complications [19]. In the present study, we demonstrated that long-term HFD intake caused significant weight gain in mice compared with the NC group, along with increased liver, gonadal adipose, and perirenal adipose tissue indexes, as well as the onset of hepatic pathological changes (e.g., lipid accumulation and hepatocyte structural damage). Additionally, HFD exposure resulted in a reduction in colon length. These observations are closely linked to the progression of HFD-induced chronic hepatic inflammation. Notably, intervention with co-fermented quinoa and black barley (FG) significantly reversed these pathological phenotypes, suggesting that FG may play a role in inhibiting the development of HFD-induced chronic hepatic inflammation. This protective effect may be attributed to bioactive components enriched in FG (e.g., ferulic acid and *p*-coumaric acid) and probiotics used in the fermentation process of FG. Our previous research revealed a notable increase in the total contents of polyphenols and flavonoids in FG compared to the unfermented counterpart [18]. Existing studies have shown that phenolic acids such as ferulic acid exhibit antioxidant, anti-inflammatory, and gut microbiota-modulating bioactivities [20,21]; however, the underlying regulatory mechanism requires further investigation.

### 4.2. FG Ameliorated Hepatic Chronic Inflammation via Regulating Hepatic Metabolites and Tlr4/Myd88/CD14 Signalling Pathway

Studies have shown that long-term intake of a HFD can induce activation of the TLR4/ MyD88/CD14 pathway in the liver. This activation promotes the release of pro-inflammatory cytokines such as TNF-α, IL-6, and IL-1β, ultimately exacerbating non-alcoholic fatty liver disease (NAFLD) [22,23]. In the present study, we found that FG intervention effectively inhibited the activation of the *Tlr4/Myd88/CD14* pathway at the transcriptional level and reduced the levels of the inflammatory cytokines TNF-α and IL-6 in liver tissue. These anti-inflammatory effects may be closely associated with the polyphenols and flavonoids enriched in FG, as well as the regulatory role of *Lactobacillus*. For instance, it has been reported that ferulic acid reduces the levels of inflammatory cytokines (TNF-α, IL-6, and IL-1β) in the liver tissue of mice with non-alcoholic steatohepatitis (NASH), ultimately alleviating liver inflammation [24]. In addition, Wang et al. [25] demonstrated that administration of *Lactobacillus* alone alleviated liver inflammation in rats with acute liver injury by downregulating the protein expression levels of TLR4, TNF-α, and nuclear factor-κB (NF-κB) in liver tissue.

To further elucidate the anti-inflammatory mechanisms of FG, this study explored the regulatory effect of FG on metabolism. We observed that FG intervention increased the levels of vitamin A and L-tryptophan while decreasing the level of taurocholic acid in the liver. Evidence indicates that VA is essential for the optimal maintenance and function of the immune system [26]. A clinical trial found that vitamin A supplementation reduced the serum levels of TNF-α and IL-6 in adults [27]. And vitamin A has been shown to suppress the mRNA expression levels of monocyte chemoattractant protein-1 (*Mcp-1*), *Cd68*, *Tnf-α*, and *Il-6* in HFD-fed mice [28]. L-tryptophan is an essential amino acid that can be converted into melatonin, 5-hydroxytryptophan, and indole compounds in the body—either through its own metabolic processes or via gut microbiota metabolism. Previous studies have shown that L-tryptophan metabolites reduce lipid accumulation in liver tissue and inhibit the mRNA expression levels of *Mcp-1* and *Tnf-α* [29,30]. In contrast, the accumulation of bile acids (BAs) in the liver can cause hepatic damage and fibrosis. Taurine cholic acid (TCA) is a primary conjugated bile acid that exists mainly in the form of bile salts. Yang et al. [31] found that under cholestatic conditions, TCA promotes the activation of hepatic stellate cells via the S1PR2/p38 MAPK/YAP pathway, ultimately leading to hepatic damage and fibrosis.

### 4.3. FG Alleviated Hepatic Inflammation via Regulating Gut Microbiota

FG alleviated hepatic inflammatory injury by regulating the gut microbiota. In the present study, we observed that FG consumption effectively reduced the Firmicutes/Bacteroidetes (F/B) ratio. Additionally, FG intervention increased the abundance of *g_Lawsonibacter* and *s_Bifidobacterium criceti* while inhibiting the proliferation of *g_Adlercreutzia*, *s_Adlercreutzia caecimuris*, and *s_Dubosiella sp004793885*.

As a well-known butyrate-producing bacterium, *g_Lawsonibacter* plays a critical role in the gut microbial community [32] and may inhibit lipid accumulation and the expression of inflammation-related cytokines [33]. A previous study demonstrated that administration of polyphenols or probiotics can promote the proliferation of *g_Lawsonibacter* [34], which in consistent with our findings. Interestingly, a recent study revealed that the abundance of *g_Lawsonibacter* decreased following weight loss in obese individuals, accompanied by significant reductions in lipid levels and plasma IL-6 concentration [35].

*s_Bifidobacterium criceti*, a member of the *Bifidobacterium* genus, is a key component of the core gut microbiota and functions as a probiotic in the intestinal ecosystems of both humans and animals. A prior study showed that *Bifidobacterium* can ameliorate hepatic steatosis and inflammation in HFD-induced chronic liver disease mice by suppressing the NF-*κ*B signaling pathway—this effect is mediated by reduced endotoxin levels and inactivation of macrophages [36].

It has been reported that the *g_Adlercreutzia* and *s_Dubosiella sp004793885* exhibit dual roles, with potential adverse (pro-inflammatory) and beneficial (anti-inflammatory) effects on health [37,38]. In the present study, the abundance of these two taxa was increased in the HFD group, implying their pro-inflammatory role in this context. Thus, the regulatory effect of FG on the growth of these bacteria may contribute to its anti-inflammatory activity.

Furthermore, KEGG functional analysis of the gut microbiota revealed that FG upregulated the BA biosynthesis pathway and the phenylalanine, tyrosine, and tryptophan (PTT) metabolism pathway (data reported in our published manuscript [39]. The PTT metabolism pathway is associated with the synthesis of tyrosine and tryptophan—precursors for the production of indole and its derivatives, such as indoleacetaldehyde and 2-indolecarboxylic acid [40]. In the current study, FG intervention significantly increased the levels of indoleacetaldehyde and 2-indolecarboxylic acid in fecal metabolites. These two metabolites have been shown to play a pivotal role in maintaining intestinal homeostasis and systemic immunity: they promote the differentiation and functional activation of anti-inflammatory macrophages, regulatory T (Treg) cells, interleukin-10 (IL-10)-producing cells, and group 3 innate lymphoid cells (ILC3s)—all of which are essential for preserving gut mucosal homeostasis [41]. A recent published study has further elaborated on the significance of indole derivatives in gut–liver axis regulation. For instance, indole-3-propionate (IPA), another important indole derivative, has been reported to be decreased in patients with inflammatory bowel disease (IBD) [42]. It plays a role in inhibiting the expression of pro-inflammatory factors in the colon and reducing the proportion of inflammatory CD4+ T cells, thereby participating in the regulation of mucosal immune responses. Another study showed that indoleacrylic acid (an indole derivative) alleviates type 2 diabetes by activating the aryl hydrocarbon receptor (AhR), which reduces IL-1β, IL-6, and TNF-α levels [43]. As a ligand-activated transcription factor, AhR transfers to the nucleus upon binding specific ligands, participating in immune regulation and other physiological functions [44]. A previous study revealed that indole-3-propionic acid treatment attenuated LPS-induced cardiac dysfunction and inflammation in rats via regulating the AhR/NF-*κ*B/NLRP3 pathway [45]. Therefore, our study implied that the increase in indoleacetaldehyde and 2-indolecarboxylic acid levels induced by FG may have a broader impact on the overall immune regulation network in the gut, not only affecting local immune cells such as macrophages and Treg cells but also potentially interacting with other components of the immune system to maintain a balanced immune state. Nevertheless, verification of this hypothesis still requires further investigation.

Moreover, the hepatic levels of BAs (taurocholic acid) were decreased after the intervention of FG. The elevated hepatic BAs will disrupt the tight junctions of biliary epithelial cells (cholangiocytes), leading to bile leakage in the periductal area, which initiates the inflammatory and fibrotic response [46]. Additionally, the decreased levels of BAs after FG intervention may also affect the gut microbiota composition. Some studies have shown that BAs can selectively inhibit the growth of certain bacteria, thus reshaping the gut microbiota community structure, which in turn may further impact host metabolism and immune function [47]. These data provide indirect evidence supporting the mechanism by which FG protects against HFD-induced hepatic inflammation.

## 5. Conclusions

In conclusion, this study investigated the beneficial role of FG in alleviating HFD-induced chronic hepatic inflammation in mice. FG exerted a foundational protective effect on HFD-induced pathological phenotypes and suppressed hepatic inflammation, as evidenced by reducing the protein concentrations of pro-inflammatory cytokines (TNF-α, IL-1β, and IL-6), and inhibiting the activation of the *Tlr4/Myd88/CD14* signaling pathway at the transcriptional level. The mechanism may involve the regulation of hepatic metabolites and the gut–liver axis. Specifically, it corrected hepatic taurocholic acid abundance and enriched gut probiotics (*g_Lawsonibacter* and *s_Bifidobacterium criceti*). This microbiota shift likely serves as an upstream event for maintaining metabolic homeostasis by shaping the fecal metabolome. The subsequent rise in indoleacetaldehyde and 2-indolecarboxylic acid levels suggests these metabolites act as signaling molecules that suppress pro-inflammatory pathways via the gut–liver axis.

However, the present study still has several limitations. First, while chronic hepatic inflammation is defined by specific phenotypic indicators, the mechanistic pathways driving these phenotypes remain poorly characterized and require further investigation. Second, the specific physiological roles of fecal and hepatic metabolites in mediating FG’s beneficial effects still need to be clarified.

## Figures and Tables

**Table 1:** Primer pairs used for the real-time quantitative PCR analysis.

Gene Name	Forward Sequence (5’ to 3’)	Reverse Sequence (5’ to 3’)
*Actb*	TTCGCGGGCGACGAT	CATCTTTTCACGGTTGGCCT
*Il-6*	GAGACTTCCATCCAGTTGCCT	TCTCCTCTCCGGACTTGTGA
*Tnf-a*	GCACCACCATCAAGGACTCA	GAGGCAACCTGACCACTCTC
*Il-1b*	GAGCACAAGCCTGTCTTCCT	TCTTGGCCGAGGACTAAGGA
*Tlr4*	GCACTGTTCTTCTCCTGCCT	AGAGGTGGTGTAAGCCATGC
*CD14*	AAGCAGATCTGGGGCAGTTC	CGCAGGGCTCCGAATAGAAT
*Myd88*	AAGCAGCAGAACCAGGAGTC	CGAAAAGTTCCGGCGTTTGT

**Table 2:** Differential hepatic metabolites altered by FG intervention.

Substance	MS (*m*/*z*)	Retention Time (min)	Mass Error (ppm)	Formula	Adducts	VIP	*p* Value	NC/HFD	HFG/HFD	HFL/HFD
Retinyl ester	303.23	9.60	−0.81	C_20_H_30_O_2_	M + H − H_2_O	2.11	5.7 × 10^−14^	↑	↑	↑
Vitamin A	269.23	9.60	−1.50	C_20_H_30_O	M + H − H_2_O	2.00	2.38 × 10^−13^	↑	↑	↑
Decanoyl-CoA	922.26	0.93	−1.35	C_31_H_54_N_7O17_P_3_S	M + H	1.09	1.36 × 10^−4^	↑	↑	↑
6-Hydroxymelatonin	271.10	0.62	−2.03	C_13_H_16_N_2_O_3_	M + Na	1.03	4.97 × 10^−4^	↑	↑	↑
L-tryptophan	227.08	3.51	−1.62	C_11_H_12_N_2_O_2_	M + Na	1.01	2.03 × 10^−8^	↑	↑	↑
Taurocholic acid	498.29	6.09	−0.82	C_26_H_45_NO_7_S	M + H − H_2_O	2.82	1.32 × 10^−15^	↓	↓	↓
Cytidine monophosphate	423.27	6.32	−1.75	C_24_H_40_O6	M − H	1.81	9.92 × 10^−10^	↑	↑	↑
1b-Hydroxycholic acid	447.27	6.55	−0.28	C_24_H_40_O_6_	M + Na	1.85	6.59 × 10^−27^	↑	↑	↑
Wharangin	362.09	2.50	−3.89	C_17_H_12_O_8_	M + NH_4_	1.61	1.62 × 10^−35^	↑	↑	↑
L-a-Lysophosphatidylcholine	490.29	7.41	−0.68	C_22_H_46_NO_7_P	M + H	2.07	3.59 × 10^−22^	↑	↑	↑
7-Methylguanine	166.07	0.93	−2.31	C_6_H_7_N_5_O	M − H	1.60	5.49 × 10^−29^	↑	↑	↑
N2-Succinyl-L-ornithine	215.10	7.63	−2.04	C_9_H_16_N_2_O_5_	M + H − H_2_O	1.76	1.03 × 10^−12^	↑	↑	↑
Luteolin 3’-glucuronide	485.07	0.64	3.81	C_21_H_18_O_12_	M + Na	2.16	4.09 × 10^−32^	↑	↑	↑
Sulfolithocholylglycine	514.28	5.75	−1.67	C_26_H_43_NO_7_S	M − H	1.96	5.72 × 10^−26^	↓	↓	↓
PE (15:0/20:2(11Z,14Z))	752.52	9.60	0.64	C_40_H_76_NO_8_P	M + Na	1.19	1.59 × 10^−4^	↑	↑	↑
N-Acetylglutamine	211.07	0.91	−2.92	C_7_H_12_N_2_O_4_	M + Na	1.70	3.57 × 10^−29^	↑	↑	↑
PC (22:5(7Z,10Z,13Z,16Z,19Z)/14:0)	802.54	7.18	1.16	C_44_H_78_NO_8_P	M + Na	1.52	2.81 × 10^−31^	↓	↓	↓

↑ increase; ↓ decrease.

**Table 3:** Differential fecal metabolites altered by FG intervention.

Substance	MS (*m*/*z*)	Retention Time (min)	Mass Error (ppm)	Formula	Adducts	VIP	*p* Value	NC/HFD	HFG/HFD	HFL/HFD
Glucosylceramide	682.46	6.98	−2.78	C_36_H_69_NO_8_	M + K	1.41	7.12 × 10^−32^	↑	↑	↑
Linoleic acid	303.23	7.38	0.72	C_18_H_32_O_2_	M + Na	1.80	2.29 × 10^−30^	↑	↑	↑
Allopregnanolone	363.25	6.24	−0.33	C_21_H_34_O_2_	M + FA − H	1.99	7.87 × 10^−8^	↑	↑	↑
Indoleacetaldehyde	160.08	2.66	−1.16	C_10_H_9_NO	M + H	2.38	2.18 × 10^−4^	↑	↑	↑
2-Indolecarboxylic acid	160.04	3.55	−2.63	C_9_H_7_NO_2_	M − H	1.69	3.57 × 10^−4^	↑	↑	↑
Chenodeoxycholic acid sulfate	945.51	5.37	−1.17	C_24_H_40_O_7_S	2M + H	1.85	9.78 × 10^−4^	↓	↓	↓
3,7-Dihydroxy-12-oxocholanoic acid	389.27	5.64	−1.70	C_24_H_38_O_5_	M + H − H_2_O	1.59	9.22 × 10^−21^	↑	↑	↑
Trigonelline	140.07	3.57	0.72	C_7_H_9_NO_2_	M + H	1.52	1.51 × 10^−10^	↑	↑	↑
Cinnamaldehyde	177.06	6.24	−2.07	C_9_H_8_O	M + FA − H	1.62	2.02 × 10^−24^	↑	↑	↑
Tolnaftate	632.24	0.77	−0.39	C_19_H_17_NOS	2M + NH_4_	1.53	3 × 10^−12^	↑	↑	↑
11-Ketoetiocholanolone	349.20	6.04	−1.83	C_19_H_28_O_3_	M + FA − H	2.43	4.27 × 10^−8^	↑	↑	↑
Disialyllactose	463.16	5.37	−2.51	C_34_H_56_N_2_O_27_	M + 2H	2.18	3.44 × 10^−21^	↑	↑	↑
11-Methyl-7-oxatetracyclo [6.3.1.01,6.04,11] dodecane	178.14	8.37	−0.49	C_12_H_18_O	M + H − H_2_O, M + H	2.26	9.06 × 10^−17^	↑	↑	↑
(3β,17α,23R)-17,23-Epoxy-3,29-dihydroxy-27-norlanost-8-ene-15,24-dione	473.33	6.59	−0.91	C_29_H_44_O_5_	M + H	1.87	3.97 × 10^−26^	↑	↑	↑
(Z)-α-Bergamotenoic acid	235.17	7.72	−1.18	C_21_H_34_O_2_	M + H	1.51	9.87 × 10^−29^	↑	↑	↑
Sebacic acid	183.10	5.57	−2.44	C_10_H_18_O_4_	M-H_2_O − H	1.62	1.04 × 10^−15^	↑	↑	↑
Amylose	160.04	3.55	−2.63	C_9_H_7_NO_2_	M − H	1.69	3.57 × 10^−4^	↑	↑	↑
O-Desmethylangolensin	257.08	5.66	−1.17	C_15_H_14_O_4_	M−H	1.64	2.32 × 10^−12^	↑	↑	↑
Tetracosatetraenoic acid n-6	395.27	6.03	−1.16	C_24_H_40_O_2_	M + Cl	1.79	3.59 × 10^−24^	↑	↑	↑
PC (18:3(6Z,9Z,12Z)/20:2(11Z,14Z))	842.55	6.83	0.24	C_46_H_82_NO_8_P	M + Cl	1.52	1.14 × 10^−10^	↓	↓	↓
LysoPC (22:6(4Z,7Z,10Z,13Z,16Z,19Z))	568.34	7.97	2.15	C_30_H_50_NO_7_P	M + H	1.76	1.03 × 10^−12^	↓	↓	↓

## Data Availability

The data is available from the corresponding author upon request due to privacy.

## Abbreviations

The following abbreviations are used in this manuscript:

HFD	high-fat diet
FG	co-fermented whole grains black barley and quinoa
TNF-α	tumor necrosis factor-α
IL-1β	interleukin-1β
IL-6	interleukin-6
TLR4	toll-like receptor 4
Myd88	myeloid differentiation primary response gene 88
CD14	cluster of differentiation 14
BA	bile acid

## References

[1] Nutter S., Eggerichs L.A., Nagpal T.S., Ramos Salas X., Chin Chea C., Saiful S., Ralston J., Barata-Cavalcanti O., Batz C., Baur L.A. (2023). Changing the global obesity narrative to recognize and reduce weight stigma: A position statement from the World Obesity Federation. Obes. Rev..

[2] Piché M.E., Tchernof A., Després J.P. (2020). Obesity Phenotypes, Diabetes, and Cardiovascular Diseases. Circ. Res..

[3] Taru V., Szabo G., Mehal W., Reiberger T. (2024). Inflammasomes in chronic liver disease: Hepatic injury, fibrosis progression and systemic inflammation. J. Hepatol..

[4] Gaggini M., Carli F., Rosso C., Buzzigoli E., Marietti M., Della Latta V., Ciociaro D., Abate M.L., Gambino R., Cassader M. (2018). Altered amino acid concentrations in NAFLD: Impact of obesity and insulin resistance. Hepatology.

[5] Kern L., Mittenbühler M.J., Vesting A.J., Ostermann A.L., Wunderlich C.M., Wunderlich F.T. (2018). Obesity-Induced TNFα and IL-6 Signaling: The Missing Link between Obesity and Inflammation-Driven Liver and Colorectal Cancers. Cancers.

[6] Zhang X., Coker O.O., Chu E.S., Fu K.L., Lau H.C.H., Wang Y.X., Chan A.W.H., Wei H., Yang X.Y., Sung J.J.Y. (2021). Dietary cholesterol drives fatty liver-associated liver cancer by modulating gut microbiota and metabolites. Gut.

[7] Cai J.W., Rimal B., Jiang C.T., Chiang J.Y.L., Patterson A.D. (2022). Bile acid metabolism and signaling, the microbiota, and metabolic disease. Pharmacol. Ther..

[8] Fan Y., Pedersen O. (2020). Gut microbiota in human metabolic health and disease. Nat. Rev. Microbiol..

[9] Sun J.Y., Fan J.M., Li T.T., Yan X.X., Jiang Y.H. (2022). Nuciferine Protects Against High-Fat Diet-Induced Hepatic Steatosis via Modulation of Gut Microbiota and Bile Acid Metabolism in Rats. J. Agric. Food Chem..

[10] Wang X., Sun Z., Wang X., Li M., Zhou B., Zhang X. (2024). Solanum nigrum L. berries extract ameliorated the alcoholic liver injury by regulating gut microbiota, lipid metabolism, inflammation, and oxidative stress. Food Res. Int..

[11] Habermaass V., Bartoli F., Gori E., Dini R., Cogozzo A., Puccinelli C., Pierini A., Marchetti V. (2024). Fecal Bile Acids in Canine Chronic Liver Disease: Results from 46 Dogs. Animals.

[12] Morigny P., Boucher J., Arner P., Langin D. (2021). Lipid and glucose metabolism in white adipocytes: Pathways, dysfunction and therapeutics. Nat. Rev. Endocrinol..

[13] Castro-Alba V., Lazarte C.E., Perez-Rea D., Carlsson N.G., Almgren A., Bergenståhl B., Granfeldt Y. (2019). Fermentation of pseudocereals quinoa, canihua, and amaranth to improve mineral accessibility through degradation of phytate. J. Sci. Food Agric..

[14] Tsafrakidou P., Michaelidou A.-M., Biliaderis C.G. (2020). Fermented Cereal-based Products: Nutritional Aspects, Possible Impact on Gut Microbiota and Health Implications. Foods.

[15] Guan Q., Ding X.-W., Zhong L.-Y., Zhu C., Nie P., Song L.-H. (2021). Beneficial effects of Lactobacillus-fermented black barley on high fat diet-induced fatty liver in rats. Food Funct..

[16] Lin Z.-H., Zhong L.-Y., Jiang H.-B., Zhu C., Wei F.-F., Wu Y., Song L.-H. (2024). Elucidation of the beneficial role of co-fermented whole grain quinoa and black barley with Lactobacillus on rats fed a western-style diet via a multi-omics approach. Food Res. Int..

[17] Zhong L.Y., Qin L.A., Ding X.W., Ma L., Wang Y., Liu M.H., Chen H., Yan H.L., Song L.H. (2022). The regulatory effect of fermented black barley on the gut microbiota and metabolic dysbiosis in mice exposed to cigarette smoke. Food Res. Int..

[18] Jiang H.B., Nie P., Lin Z.H., Zhu C., Zhong L.Y., Wei F.F., Wu Y., Song L.H. (2024). The polyphenols profile of co-fermented quinoa and black barley with Lactobacillus kisonensis and its in vitro bioactivities. Food Biosci..

[19] Dicken S.J., Batterham R.L. (2021). The Role of Diet Quality in Mediating the Association between Ultra-Processed Food Intake, Obesity and Health-Related Outcomes: A Review of Prospective Cohort Studies. Nutrients.

[20] Mei Z., Hong Y., Yang H., Cai S., Hu Y., Chen Q., Yuan Z., Liu X. (2023). Ferulic acid alleviates high fat diet-induced cognitive impairment by inhibiting oxidative stress and apoptosis. Eur. J. Pharmacol..

[21] Li D., Rui Y.-x., Guo S.-d., Luan F., Liu R., Zeng N. (2021). Ferulic acid: A review of its pharmacology, pharmacokinetics and derivatives. Life Sci..

[22] Zhang Y., He X., Wang K., Xue Y., Hu S., Jin Y., Zhu G., Shi Q., Rui Y. (2024). Irisin alleviates obesity-induced bone loss by inhibiting interleukin 6 expression via TLR4/MyD88/NF-κB axis in adipocytes. J. Adv. Res..

[23] Liu Y., Tian Y., Dai X., Liu T., Zhang Y., Wang S., Shi H., Yin J., Xu T., Zhu R. (2023). Lycopene ameliorates islet function and down-regulates the TLR4/MyD88/NF-κB pathway in diabetic mice and Min6 cells. Food Funct..

[24] Wei Z., Xue Y., Xue Y., Cheng J., Lv G., Chu L., Ma Z., Guan S. (2021). Ferulic acid attenuates non-alcoholic steatohepatitis by reducing oxidative stress and inflammation through inhibition of the ROCK/NF-κB signaling pathways. J. Pharmacol. Sci..

[25] Wang Q.Q., Lv L.X., Jiang H.Y., Wang K.C., Yan R., Li Y.T., Ye J.Z., Wu J.J., Wang Q., Bian X.Y. (2019). R0052 alleviates liver injury by modulating gut microbiome and metabolome in d-galactosamine-treated rats. Appl. Microbiol. Biot..

[26] Amimo J.O., Michael H., Chepngeno J., Raev S.A., Saif L.J., Vlasova A.N. (2022). Immune Impairment Associated with Vitamin A Deficiency: Insights from Clinical Studies and Animal Model Research. Nutrients.

[27] Gholizadeh M., Basafa Roodi P., Abaj F., Shab-Bidar S., Saedisomeolia A., Asbaghi O., Lak M. (2022). Influence of Vitamin A supplementation on inflammatory biomarkers in adults: A systematic review and meta-analysis of randomized clinical trials. Sci. Rep..

[28] Melnikov N., Kamari Y., Kandel-Kfir M., Barshack I., Ben-Amotz A., Harats D., Shaish A., Harari A. (2022). β-Carotene from the Alga Dunaliella bardawil Decreases Gene Expression of Adipose Tissue Macrophage Recruitment Markers and Plasma Lipid Concentrations in Mice Fed a High-Fat Diet. Mar. Drugs.

[29] Fiore A., Murray P.J. (2021). Tryptophan and indole metabolism in immune regulation. Curr. Opin. Immunol..

[30] Ji Y., Gao Y., Chen H., Yin Y., Zhang W. (2019). Indole-3-Acetic Acid Alleviates Nonalcoholic Fatty Liver Disease in Mice via Attenuation of Hepatic Lipogenesis, and Oxidative and Inflammatory Stress. Nutrients.

[31] Yang J., Tang X., Liang Z., Chen M., Sun L. (2023). Taurocholic acid promotes hepatic stellate cell activation via S1PR2/p38 MAPK/YAP signaling under cholestatic conditions. Clin. Mol. Hepatol..

[32] Sakamoto M., Iino T., Yuki M., Ohkuma M. (2018). Lawsonibacter asaccharolyticus gen. nov., sp. nov., a butyrate-producing bacterium isolated from human faeces. Int. J. Syst. Evol. Microbiol..

[33] Fang W., Xue H., Chen X., Chen K., Ling W. (2019). Supplementation with Sodium Butyrate Modulates the Composition of the Gut Microbiota and Ameliorates High-Fat Diet-Induced Obesity in Mice. J. Nutr..

[34] Le Sayec M., Xu Y., Laiola M., Gallego F.A., Katsikioti D., Durbidge C., Kivisild U., Armes S., Lecomte M., Fança-Berthon P. (2022). The effects of Aronia berry (poly)phenol supplementation on arterial function and the gut microbiome in middle aged men and women: Results from a randomized controlled trial. Clin. Nutr..

[35] Prykhodko O., Burleigh S., Campanello M., Iresjö B.-M., Zilling T., Ljungh Å., Smedh U., Hållenius F.F. (2024). Long-Term Changes to the Microbiome, Blood Lipid Profiles and IL-6 in Female and Male Swedish Patients in Response to Bariatric Roux-en-Y Gastric Bypass. Nutrients.

[36] Min B.H., Devi S., Kwon G.H., Gupta H., Jeong J.J., Sharma S.P., Won S.M., Oh K.K., Yoon S.J., Park H.J. (2024). Gut microbiota-derived indole compounds attenuate metabolic dysfunction-associated steatotic liver disease by improving fat metabolism and inflammation. Gut Microbes.

[37] Clos-Garcia M., Garcia K., Alonso C., Iruarrizaga-Lejarreta M., D’Amato M., Crespo A., Iglesias A., Cubiella J., Bujanda L., Falcón-Pérez J.M. (2020). Integrative Analysis of Fecal Metagenomics and Metabolomics in Colorectal Cancer. Cancers.

[38] Xu X., Li G., Zhang D., Zhu H., Liu G.h., Zhang Z. (2023). Gut Microbiota is Associated with Aging-Related Processes of a Small Mammal Species under High-Density Crowding Stress. Adv. Sci..

[39] Wei F.F., Jiang H.B., Zhu C., Zhong L.Y., Lin Z.H., Wu Y., Song L.H. (2024). The co-fermentation of whole-grain black barley and quinoa improves murine cognitive impairment induced by a high-fat diet via altering gut microbial ecology and suppressing neuroinflammation. Food Funct..

[40] Rovelli V., Longo N. (2023). Phenylketonuria and the brain. Mol. Genet. Metab..

[41] Su X., Gao Y., Yang R. (2022). Gut Microbiota-Derived Tryptophan Metabolites Maintain Gut and Systemic Homeostasis. Cells.

[42] Gao H., Sun M., Li A., Gu Q., Kang D., Feng Z., Li X., Wang X., Chen L., Yang H. (2025). Microbiota-derived IPA alleviates intestinal mucosal inflammation through upregulating Th1/Th17 cell apoptosis in inflammatory bowel disease. Gut Microbes.

[43] Liu D., Zhang S., Li S., Zhang Q., Cai Y., Li P., Li H., Shen B., Liao Q., Hong Y. (2023). Indoleacrylic acid produced by Parabacteroides distasonis alleviates type 2 diabetes via activation of AhR to repair intestinal barrier. BMC Biol..

[44] Polonio C.M., McHale K.A., Sherr D.H., Rubenstein D., Quintana F.J. (2025). The aryl hydrocarbon receptor: A rehabilitated target for therapeutic immune modulation. Nat. Rev. Drug Discov..

[45] Zhang Y., Li S., Fan X., Wu Y. (2024). Pretreatment with Indole-3-Propionic Acid Attenuates Lipopolysaccharide-Induced Cardiac Dysfunction and Inflammation Through the AhR/NF-κB/NLRP3 Pathway. J. Inflamm. Res..

[46] Zeng J., Fan J.A., Zhou H.P. (2023). Bile acid-mediated signaling in cholestatic liver diseases. Cell Biosci..

[47] Kiriyama Y., Nochi H. (2019). The Biosynthesis, Signaling, and Neurological Functions of Bile Acids. Biomolecules.

